# Short communication: Mpox memes, the gift that conceals a blade

**DOI:** 10.1371/journal.pgph.0004496

**Published:** 2025-05-07

**Authors:** Ivaan Pitua, Raafidha Raizudheen

**Affiliations:** Makerere University, College of Health Sciences, Kampala, Uganda; McGill University, CANADA

## Abstract

Memes have become a popular communication tool, especially during public health crises, offering both opportunities and challenges for messaging. This short communication examines the dual role of mpox-related memes circulating since the disease’s declaration as a public health emergency in August 2024. Through a qualitative content analysis of 200 mpox memes shared across major social media platforms, we identified two primary themes: (1) Awareness and Education and (2) Misinformation and Stigma. Approximately 60% of the memes promoted awareness, using humor to increase engagement with health messages and fostering constructive dialogue on preventive measures. However, 40% conveyed misinformation or perpetuated stigma, particularly against LGBTQ+ communities, which may have hindered public trust in health authorities and created barriers to health-seeking behaviors. The findings suggest that while memes can positively impact public health communication, their potential to spread misinformation requires strategic monitoring and response. Public health organizations may benefit from partnering with social media influencers to create accurate, relatable meme content that engages audiences without perpetuating harmful stereotypes.

## Introduction

Mpox is a viral disease that is usually zoonotic. The disease is caused by the Monkeypox virus, which is present in the wildlife in several central- and west-African countries [1]. In August 2024, the Africa Centres for Disease Control and Prevention and the World Health Organization declared mpox a Public Health Emergency of Continental Security and a Public Health Emergency of International Concern, respectively [2], which was greeted with a wave of reactions from the public. Memes have become a widely used form of communication, especially during health crises such as the present mpox outbreak [3]. While they offer a unique opportunity for raising awareness, they also pose risks related to misinformation and stigmatization. As noted during the Coronavirus disease 2019 (COVID19) pandemic, memes have been a mainstay, the public uses it for coping, maintaining humor, sharing information about the disease and also to resist or defy public health communications [4,5]. We explored the dual role of mpox memes in public health and examined how they can both help and harm public health messaging.

## Materials and methods

A qualitative content analysis was conducted on mpox-related memes with English-language content circulating across major different media platforms, specifically focusing on Google, X (formerly Twitter), Instagram, Reddit, Tiktok, and Facebook. Memes were identified using the hashtag #mpox, a google search with the term “mpox memes” returned a compilation of memes shared in the different social media platforms. Healthcare professionals were identified based on self-reported information in their profile bios. Secondary validation through professional registrations or institutional affiliations was not performed. A sample of 200 memes was selected from posts between August, 2024 when mpox was declared a public health emergency of international concern and 31^st^ October, 2024. We followed the memes on google to check when they were shared before including in analysis. Where retrieved posts had no memes, we used snowballing to collect memes from the comments and comment reply section. While these memes represent diverse users, the sample predominantly reflects English-speaking regions. No systematic cross-regional comparisons were performed. The memes were coded for themes of awareness promotion, humor, misinformation, and stigma. The collection and analysis of all mpox memes complied with the terms of service for each platform on the use of public content. Individual user names, links to social media page or links to particular memes are not included in the article metadata to avoid breach of privacy as certain contents shared were sensitive. Metadata for all 200 memes have been provided as supplementary information, S1 Data.

## Results

A total of 200 mpox-related memes were analyzed, revealing two primary themes: (1) Awareness and Education, and (2) Misinformation and Stigma. Within these themes, sub-themes emerged that provided further insights into how memes influenced public perception and behavior regarding mpox.

### 3.1 Awareness and education

Most memes (60%) aimed to inform audiences about mpox symptoms, preventive measures, and testing locations, often using humor or relatable scenarios to make the information more accessible and engaging. Sub-themes included:

#### 3.1.1 Symptom recognition.

Approximately 25% of the awareness-oriented memes depicted common mpox symptoms in a humorous way, using exaggerated imagery or captions that helped viewers remember signs of the disease. For example, some memes humorously compared mpox rashes to common skin conditions, which seemed to reduce anxiety around symptom recognition and prompted users to seek testing when necessary based on the comments.

#### 3.1.2 Prevention and safety measures.

Memes focusing on preventive measures, such as vaccination and hygiene practices, made up around 20% of this category. These memes often used light-hearted jokes to destigmatize discussions around vaccination. Users frequently shared these memes, tagging friends or family members to encourage safer health practices.

#### 3.1.3 Trusted voices and professional input.

In 15% of the awareness memes, medical professionals or health agencies were referenced or humorously incorporated. Such memes often gained attention from healthcare professionals, who engaged in comments or even re-shared them, lending credibility to the messages.

#### 3.1.4 Impact on public health behavior.

Awareness-based memes appeared to contribute positively to health-seeking behaviors. An observed increase in user comments indicated that these memes encouraged some users to ask questions or share personal experiences related to mpox symptoms.

### 3.2 Misinformation and stigma

A substantial portion (40%) of the memes circulated misinformation or perpetuated stigmatization of specific groups, such as the LGBTQ+ community. This theme was further divided into:

#### 3.2.1 Exaggeration and fearmongering.

Roughly 20% of these memes amplified the perceived severity of mpox, using sensational language or alarming visuals that suggested the disease was either uncontainable or far more lethal than reported. These memes often generated high engagement, with users sharing them widely and expressing heightened concern.

#### 3.2.2 Targeted stigmatization.

Another 15% of memes included stigmatizing language or imagery targeting specific demographics, particularly the LGBTQ+ community. Such memes reinforced harmful stereotypes, leading to discussions that often contained prejudiced or derogatory remarks.

#### 3.2.3 Conspiracy theories and misinformation.

Around 5% of memes endorsed conspiracy theories related to mpox origins, such as claims of government or pharmaceutical agendas. These memes, although fewer, were shared widely, often generating distrust in public health measures. Engagement with these memes typically involved users expressing distrust or sharing similar conspiracy beliefs, potentially undermining public trust in mpox-related health guidance.

## Discussion

Our analysis shows that mpox memes have a mixed impact on public health communication. While 60% of the memes effectively promoted awareness through humor, engaging users and encouraging health-seeking behaviors, 40% spread misinformation or stigma, especially against marginalized groups like the LGBTQ+ community. This stigmatization could hinder affected individuals from accessing health services due to fear of discrimination. Humor-based memes had high engagement, suggesting that public health campaigns could benefit from adopting similar formats, balancing humor with factual information to reinforce positive health behaviors. Such engagement suggests that memes promoting awareness may support public health goals by fostering dialogue around health behaviors. However, memes that conveyed exaggerated fears or conspiracy theories risked amplifying public distrust in health authorities. To counter these effects, public health agencies might consider partnering with social media influencers to create accurate, relatable meme content [6]. Platforms could also help by flagging or contextualizing harmful content, ensuring that health messages reach audiences without fueling stigma or fear [7]. More so, anti-vaccination campaigns are often shared during such public health concerns causing very huge outcries in public perception and reception for the intervention [8]. The findings primarily reflect meme trends within English-speaking regions, limiting insights into cultural or regional variations in meme usage. Humor and messaging styles are often culturally specific, and regional factors could influence how memes are created, shared, and interpreted. Future research should explore cross-regional differences to better understand the global dynamics of health-related meme communication. Such analyses could inform tailored public health messaging strategies that resonate across diverse cultural contexts.

Despite our focus on only English-language content, this study highlights the potential of memes as both tools for engagement and sources of misinformation. Future studies should explore these dynamics across languages and cultural contexts to better inform strategies for effective health communication.

## Conclusion

While mpox-related memes have a significant potential to both positively influence public health behaviors and spread misinformation, their impact on public health is largely shaped by the tone and content. Public health campaigns should carefully consider the benefits and drawbacks of meme culture, leveraging humor and relatability to promote accurate information without reinforcing harmful stereotypes or normalizing undesirable behaviors such as trolling. We recommend three strategies; Accurate Information, Addressing Stigma, Engagement with Humor by collaborating with health professionals to ensure the accuracy of content, avoiding content that targets specific groups, especially marginalized communities, and careful use of humor to attract attention while conveying factual health messages respectively.

## Supporting information

S1 Data Raw data. (XLSX)
